# A randomized trial to evaluate the pharmacokinetics, pharmacodynamics, and safety of vadadustat in patients with anemia associated with chronic kidney disease receiving hemodialysis

**DOI:** 10.1186/s12882-025-04367-x

**Published:** 2025-08-11

**Authors:** Pamela Navarro-Gonzales, Ajit Chavan, Don Wang, Steven K. Burke, Kevin Dykstra

**Affiliations:** Akebia Therapeutics, Inc, 245 1st St, Cambridge, MA 02142 USA

**Keywords:** Vadadustat, HIF-PHI, Phase 1, Pharmacokinetics, Pharmacodynamics, Safety

## Abstract

**Background:**

Vadadustat is an oral hypoxia-inducible factor prolyl hydroxylase inhibitor for treatment of anemia in dialysis-dependent chronic kidney disease (CKD) with a starting dose of 300 mg once daily (dose adjustments up to 600 mg). A recent phase 1b study evaluated the pharmacokinetics, pharmacodynamics, and safety of higher vadadustat doses (500–900 mg) in healthy volunteers. Here we report the pharmacokinetic (PK), pharmacodynamic (PD), and safety characterization of higher doses of vadadustat in patients with CKD receiving dialysis.

**Methods:**

This phase 1b, randomized, open-label study evaluated the pharmacokinetics and pharmacodynamics of vadadustat (600, 750, or 900 mg) in patients with CKD-related anemia receiving hemodialysis over a 10-day treatment period. Forty-six eligible patients were randomized to vadadustat 600, 750, or 900 mg daily or an intravenous erythropoiesis-stimulating agent. For vadadustat groups, blood samples for PK and PD analyses were collected on Day 1 and Day 8. PK analyses included area under the plasma concentration time curve (AUC) from dosing to last quantifiable concentration and to infinity, and to maximum plasma concentration (C_max_). PD analyses measured serum erythropoietin (EPO), hemoglobin, and red blood cells (RBCs). Safety assessments included adverse events in the safety population (patients who received ≥ 1 dose of study drug). Patients underwent a 30-day safety follow-up period after the last dose of study drug.

**Results:**

In the vadadustat groups, a dose-dependent increase in plasma exposure of vadadustat (C_max_ and AUC) with modest accumulation was observed on Day 1 and Day 8. Vadadustat increased plasma EPO concentrations, with a variable EPO response observed in each group. Relative to baseline, mean hemoglobin and RBC levels remained unchanged, with no significant changes observed in any treatment group. Vadadustat was welltolerated.

**Conclusions:**

The current study characterized the PK and PD response (EPO and reticulocytes) and safety profile of vadadustat at doses of 600, 750, and 900 mg in patients with CKD receiving dialysis. Overall, vadadustat was well tolerated. These findings will contribute to the development of higher-dose regimens for further investigation in phase 3 studies.

**Trial Registration:**

ClinicalTrials.gov ID NCT03992066; https://clinicaltrials.gov/study/NCT03992066; Retrospectively registered on June 18, 2019. Accessed January 13, 2025.

**Supplementary information:**

The online version contains supplementary material available at 10.1186/s12882-025-04367-x.

## Introduction

Anemia is a frequent complication in patients with chronic kidney disease (CKD). Anemia may develop early in the course of CKD but its severity increases with the decline of renal function [1]. Although the pathogenesis of anemia in CKD is multifactorial, deficiency of erythropoietin (EPO) production is a predominant cause, as the kidneys are unable to secrete sufficient EPO to support erythropoiesis and maintain hemoglobin within normal ranges [2, 3]. The current standard of care for the treatment of anemia in CKD is use of erythropoiesis-stimulating agents (ESAs) alone or in combination with iron supplementation [4]. However, ESAs are associated with increased risk of cardiovascular and thromboembolic events [5, 6]. Furthermore, ESAs require parenteral administration and refrigeration. Thus, oral treatment modalities for anemia associated with CKD may be beneficial [7–9].

Hypoxia-inducible factor prolyl hydroxylase inhibitors (HIF-PHIs) are an oral treatment alternative for patients with CKD-related anemia [7, 10]. HIF-PHIs have been shown to stabilize HIF, a transcription factor that regulates hypoxia-sensitive genes, stimulating EPO production, with a resultant increase in red blood cell (RBC) production as well as improved iron uptake and utilization [10].

Vadadustat, an orally bioavailable HIF-PHI, is approved in 37 countries globally for the treatment of CKD-related anemia in dialysis-dependent (DD) patients. It has a starting dose of 300 mg once daily and dose titration in increments or decrements of 150 mg within the range of 150 to 600 mg to achieve or maintain hemoglobin levels within the clinical target range [11, 12]. In Japan, vadadustat is approved in both non–DD (NDD)-CKD and DD-CKD populations [13, 14].

In previous clinical studies, vadadustat has been shown to maintain and increase mean hemoglobin levels within prespecified physiological target ranges [11, 12, 15, 16]. In addition, vadadustat has been shown to increase endogenous EPO production, improve iron availability, and increase circulating reticulocytes in patients with CKD and anemia [17].

Early non-clinical and clinical studies have shown that vadadustat is primarily metabolized through glucuronidation, resulting in vadadustat-O-glucuronide, which is an inactive metabolite [18]. In healthy individuals, vadadustat and its main metabolite are eliminated from the body through both renal and fecal routes. Vadadustat has also exhibited dose-proportional kinetics and rapid absorption, with a median time to maximum concentration (T_max_) of 3 to 4 hours for doses ranging from 80 to 1200 mg. Vadadustat plasma half-life (t_1/2_) is around 4 to 5 hours in healthy volunteers. However, in patients with CKD stages 3 and 4 who are not yet on dialysis, there was a modest increase in t_1/2_ from 4.2 hours to 7.9 hours, and a decrease in oral clearance (CL/F) from 1.5 L/h to 1.0 L/h [19]. Here, we report the pharmacokinetic (PK), pharmacodynamic (PD), and safety characterization of higher daily doses of vadadustat (600, 750, and 900 mg) in patients with CKD who are receiving dialysis. Treatment with vadadustat was compared with an active control group in which patients continued their current ESA treatments of either intravenous (IV) darbepoetin alfa or epoetin alfa.

## Methods

### Trial design

**Fig. 1:**
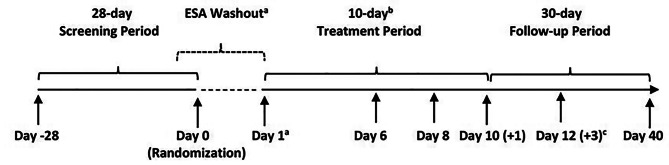
Study design. (**a**) Patients in the vadadustat groups did not receive a dose of ESA following randomization or for the duration of the 10-day treatment period. The ESA washout period started from the time of the last ESA dose prior to randomization, which may have included day of randomization. (**b**) Patients began their 10-day treatment period within 36 days of initiating screening. Day 1 occurred after a short interdialytic period at the earliest in-clinic visit following randomization. (**c**) Patients who completed the 10-day treatment period had a follow-up visit 2 days (+3) after Day 10. Patients who discontinued treatment early had a follow-up visit 2 days (+3) after their end-of-treatment visit. ESA, erythropoiesis-stimulating agent

This was a phase 1b, randomized, open-label study to evaluate the pharmacokinetics and pharmacodynamics of vadadustat 600, 750, or 900 mg daily in patients with CKD-related anemia receiving hemodialysis conducted at 10 study sites in the United States between June 2019 and May 2020. The study included a 28-day Screening and Randomization period, a 10-day Treatment Period, and a 30-day Safety Follow-up Period after the last dose of study drug. Eligible patients were randomized 2:2:2:1 to vadadustat 600, 750, or 900 mg daily, or IV ESA (epoetin alfa or darbepoetin alfa). Enrollment was handled by the site coordinator; randomization and assignment were implemented by Firma Clinical Research. The randomization process was intended to be performed using an electronic data capture system, but due to the small planned sample size of 35 PK-evaluable patients with 2 stratification factors (ESA type and average ESA dose over 8 weeks prior to randomization), a semi-electronic approach with continuous manual review was used for randomization. This allowed for participants who were not PK evaluable to be replaced during the study. With a block size of 7, there were 10 blocks for each of the 2 baseline stratification factors. For patients randomized to vadadustat, study duration also included a minimum 5- or 9-day ESA washout period prior to dosing on Day 1 (Fig. 1). On Day 1 and Day 8, participants took vadadustat after all predose procedures were completed and within 30 minutes prior to the start of dialysis so that the 1-hour postdose blood samples for PK and PD were collected during dialysis. Vadadustat was provided as 150 mg white or off-white round, biconvex, film-coated tablets for oral administration.

### Study population

Patients eligible for this study were aged ≥ 18 years at the time of informed consent confirmation and had stable baseline hemoglobin values between 8.5 and 10.5 g/dL based on 2 average values obtained within 28 days prior to randomization. Study participants were required to be on a stable 3 times weekly hemodialysis regimen, with adequate dialysis clearance defined as a single-pool Kt/Vurea of ≥ 1.2 for at least 12 weeks prior to screening. “K” is dialyzer urea clearance, “t” is total dialysis session time, and “V” is volume of distribution of urea, which is approximately equal to total body water. Study participants were also required to be maintained on IV ESA therapy with a mean dose of < 1.5 μg/kg/week for darbepoetin alfa, or mean dose of < 300 U/kg/week for epoetin alfa for 8 weeks prior to randomization. Patients were ineligible for the study if they had an RBC transfusion within 8 weeks prior to randomization. Patients with low values of ferritin (<200 ng/mL), transferrin saturation (<15%), folate (<5.5 ng/mL), or vitamin B12 (<211 pg/mL) were also ineligible. Additional exclusion criteria included a planned change in dialysis modality or renal transplant within the study period; significant cardiovascular, hematologic, hepatic, or chronic inflammatory disease; active or recent gastrointestinal (GI) bleeding; history of new or recurrent malignancy within 2 years prior to screening; currently receiving treatment or suppressive therapy for cancer. Written informed consent was obtained from all participants.

### Vadadustat pharmacokinetic assessments

For each vadadustat treatment group, blood samples for vadadustat PK analysis were collected pre-dose and at 1, 2, 3, 4, 5, 8, 11, and 24 hours post-dose on Day 1 and at pre-dose and at 1, 2, 3, 4, 5, 8, and 11 hours post-dose on Day 8. Primary endpoints were area under the plasma concentration time curve (AUC) from dosing to last quantifiable concentration (AUC_0-last_) and to infinity (AUC_0-inf_), as well as maximum plasma concentration (C_max_). Additional PK parameters included T_max_, clearance (CL/F), volume of distribution (Vz), and elimination half-life (t_½_).

PK parameters were calculated using an SAS^®^ program (SAS Institute Inc.), which followed the same algorithm as that implemented in WinNonlin^®^ Professional (Version 7.0, Pharsight Corporation, a Certara Company) using the PK population (all patients completing the study without any protocol deviation[s] that would impact the PK profile characterization). Additionally, the SAS results were validated using the parameters generated from WinNonlin 7.0. C_max_ and T_max_ were determined directly from the concentration time profiles. For AUC calculations, the entire 24-hour sampling period was included for calculating PK parameters on Day 1 using the linear up log down calculation method. AUC_0-last_ is equivalent to AUC from time 0 to 24 hours (AUC_0-24_) on Day 1. The pre-dose sample obtained on Day 8 was used as the 24-hour time point on the same day for calculating the AUC_0-24_ (which is equivalent to AUC_inf_) under the assumption that vadadustat had reached steady state by that day. Apparent clearance (CL/F) and apparent volume of distribution (Vz/F) were calculated using noncompartmental analysis. Specifically, on Day 1, CL/F and Vz/F were determined using AUC_inf_. On Day 8, following multiple dosing, CL/F and Vz/F were calculated using AUC_0–24_.

### Pharmacodynamic assessments

For each vadadustat treatment group, blood samples for EPO analysis were collected at pre-dose and at 1, 2, 3, 4, 5, 8, 11, and 24 hours post-dose on Day 1 and at pre-dose and at 1, 2, 3, 4, 5, 8, and 11 hours post-dose on Day 8. Blood samples for other PD biomarkers, hemoglobin, and RBCs were collected at pre-dose on Days 1, 6, 8, 10, and 12 (follow-up visit). Reticulocyte samples were collected on Days 1, 6, 8, and 10. PD biomarker concentrations were summarized by dose level and dosing day using descriptive statistics and graphical displays citing the PK population. For the ESA treatment group, blood samples for analysis of EPO were collected at Day 1 at pre-dose, and 0.25, 1, 2, 3, 4, 5, 8, 11, and 24 hours post-dose.

### Bioanalysis of samples

The concentration measurements of vadadustat were performed by a validated liquid chromatography-mass spectrometry/mass spectrometry method at Syneos Health Clinic (Quebec, Canada) in stabilized human EDTA K_2_ plasma with a lower limit of quantification of 100 ng/mL. The between-run (interday) accuracy ranged from 1.28% to 3.50% bias, with precision (CV%) between 2.52% and 5.22%. Within-run (intraday) accuracy showed a bias range of − 3.20% to 8.92%, with precision between 1.41% and 5.35%. All values were within the acceptance criteria of ± 15% for accuracy and ≤ 15% CV for precision, indicating an acceptable method performance. The EPO measurements were performed in serum by a sandwich enzyme-linked immunoassay (Quantikine IVD EPO ELISA; R&D Systems, Minneapolis, MN) at Q2 Solutions (Marietta, GA) [20]. Other PD endpoints (reticulocytes, RBCs, and hemoglobin) were tested at the central lab facility (ACM Medical Laboratory, Rochester, NY) using existing validated methods. All study samples were analyzed within analytical runs that complied with the acceptance ranges for the quality control samples and were completed within the known period of analyte stability.

### Safety assessment

Safety assessments included adverse events (AEs), vital signs, clinical laboratory values, and physical examinations in the safety population (all patients who received at least 1 dose of study drug). An AE that started during the treatment period or up to 30 days after the last dose of study drug was considered a treatment-emergent AE (TEAE) if it was not present prior to the first dose of study drug, or it was present prior to the first dose of study drug but increased in severity during the treatment period or up to 30 days after the last dose of study drug. AEs of special interest were also assessed, including, but not limited to, malignancy, elevation in alanine aminotransferase (ALT) or aspartate aminotransferase (AST) liver enzymes > 3 times the upper limit of normal, and pulmonary hypertension. All AEs were coded using Medical Dictionary for Regulatory Activities Version 23.0.

### Statistics

Data collected throughout the study were summarized using descriptive statistics by treatment group and listed by patient. Due to the exploratory nature of this study, no formal power analysis was conducted. Ten patients per group for the vadadustat groups and 5 patients for the ESA group were planned and an additional 11 patients were randomized to account for patients who may not have evaluable PK data.

## Results

### Trial population

A summary of patient demographic characteristics is provided for the randomized population in Table A1; patient disposition (Fig. A1) and analysis populations (Table A2) are also included in additional file 1. The treatment groups were well balanced regarding mean age, ethnicity, race, sex, height, and weight between all vadadustat groups and the IV ESA treatment group. This was similar for the safety and PK populations (data not shown). In the 750-mg dose group, 1 patient had inadvertent ESA administration and another patient had the entire concentration profile below quantitation limit for every vadadustat PK sample and therefore, these 2 patients were excluded from the PK population.

### Pharmacokinetic results

**Fig. 2:**
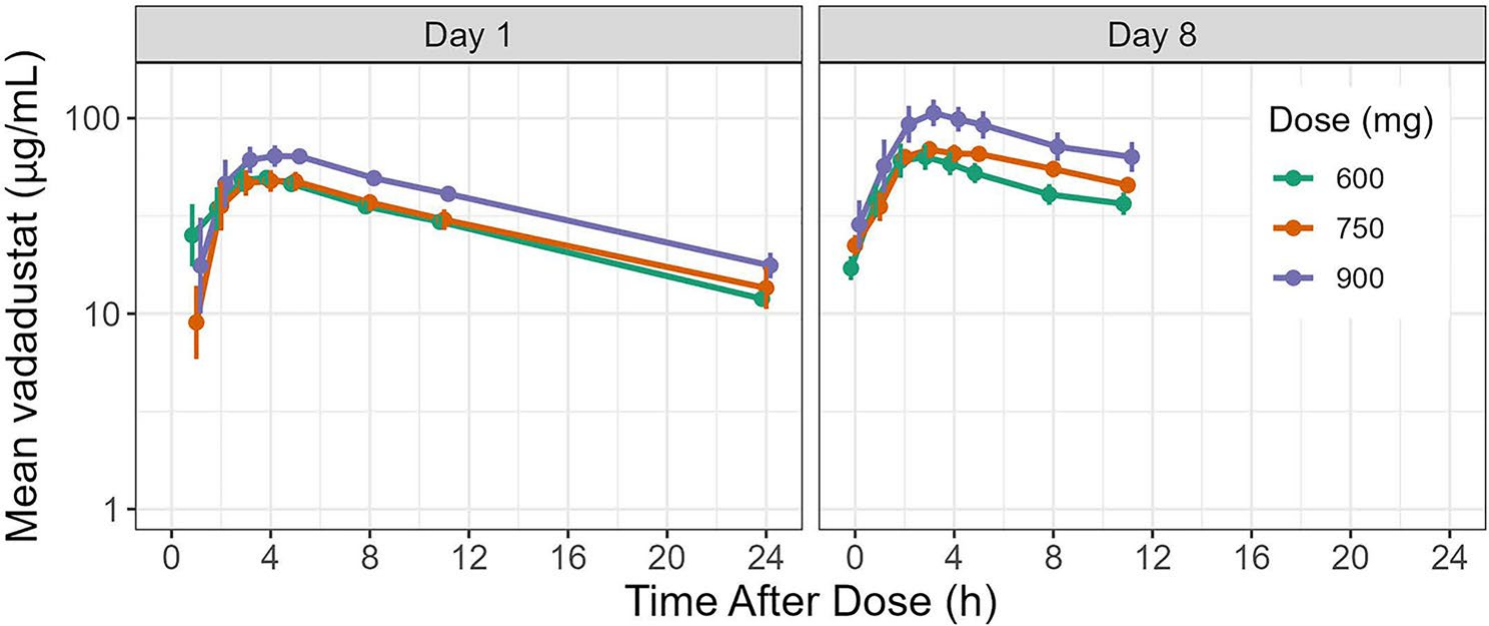
Mean plasma concentration-time profile for vadadustat (µg/mL) on Day 1 and Day 8. Error bars indicate 95% CI; geometric mean values were used for vadadustat concentration calculations. Samples were collected on Day 1 and Day 8 during dialysis. h, hours

Plasma concentration-time profiles of vadadustat after oral administration are shown in Fig. 2. After the first day of dosing, a rise in vadadustat exposure was observed as measured by AUC that corresponded with an increase in dose. This dose-dependent increase was consistently seen on both Day 1 and Day 8, as evidenced by the elevated C_max_ and AUC values. Mean values of C_max_ and AUC_0-24_ were higher on Day 8 compared with Day 1, indicating minimal accumulation of vadadustat; ratios for Day 8/Day 1 of mean C_max_ values ranged from 1.27 to 1.45, and ratios of mean AUC_0-24_ values ranged from 1.39 to 1.84, with the 900-mg dose having the highest ratios. Mean apparent total body clearance (CL/F) values for each dose on Day 8 were similar, consistent with the dose-proportional pharmacokinetics observed previously after single and multiple doses [19]. The median T_max_ of vadadustat was between 2.0 and 3.0 hours, and appeared to be similar across the 600-, 750-, and 900-mg vadadustat treatment groups when measured on Day 1 and Day 8 during dialysis (Table 1).

**Table 1:** Summary of pharmacokinetic parameters for plasma vadadustat concentration for vadadustat treatment groups

Parameter (Unit)	Statistic	Vadadustat 600 mg, N = 12		Vadadustat 750 mg, N = 10		Vadadustat 900 mg, N = 13	
		Day 1	Day 8	Day 1	Day 8	Day 1	Day 8
C_max_ (μg/mL)	Mean (SD)	62.9 (21.5)	80.9 (38.1)	67.5 (27.0)	85.5 (24.7)	88.7 (31.2)	129 (65.9)
AUC_0-24_ (h · μg/mL)	Mean (SD)	679 (174.0)	947 (440.1)	779 (398.7)	1099 (263.5)	968 (331.6)	1781 (1197)
t_1/2_ (h)	Mean (SD)	10.4 (2.3)	12.2 (3.8)	9.9 (2.8)	16.4 (8.6)	12.6 (5.1)	21.7 (26.0)
T_max_ (h)	Median (min, max)	3.0 (1.0, 5.0)	2.0 (1.0, 5.0)	3.0 (1.0, 23.8)	3.0 (1.0, 8.0)	3.1 (1.0, 8.0)	3.0 (1.0, 4.1)
CL/F (L/h)	Mean (SD)	0.8 (0.2)	0.7 (0.3)	1.3 (0.6)	0.7 (0.2)	1.0 (0.1)	0.8 (0.5)
Vz/F (L)	Mean (SD)	9.8 (1.6)	11.8 (5.4)	14.0 (6.8)	16.9 (7.8)	12.8 (2.2)	13.2 (6.0)

Day 8 included samples collected from pre-dose to 11 hours post-dose of Day 8 to calculate C_max_ and T_max_. In addition, Day 8 pre-dose was considered as a result for the 24-hour time point to calculate AUC_0-24_, t_1/2_, CL/F, and Vz/F. AUC_0-24_, area under concentration-time curve from time 0 to 24 hours; CL/F, oral clearance; C_max_, maximum observed plasma concentration; t_1/2_, half-life; T_max_, time to maximum concentration; Vz/F, volume of distribution.

**Fig. 3:**
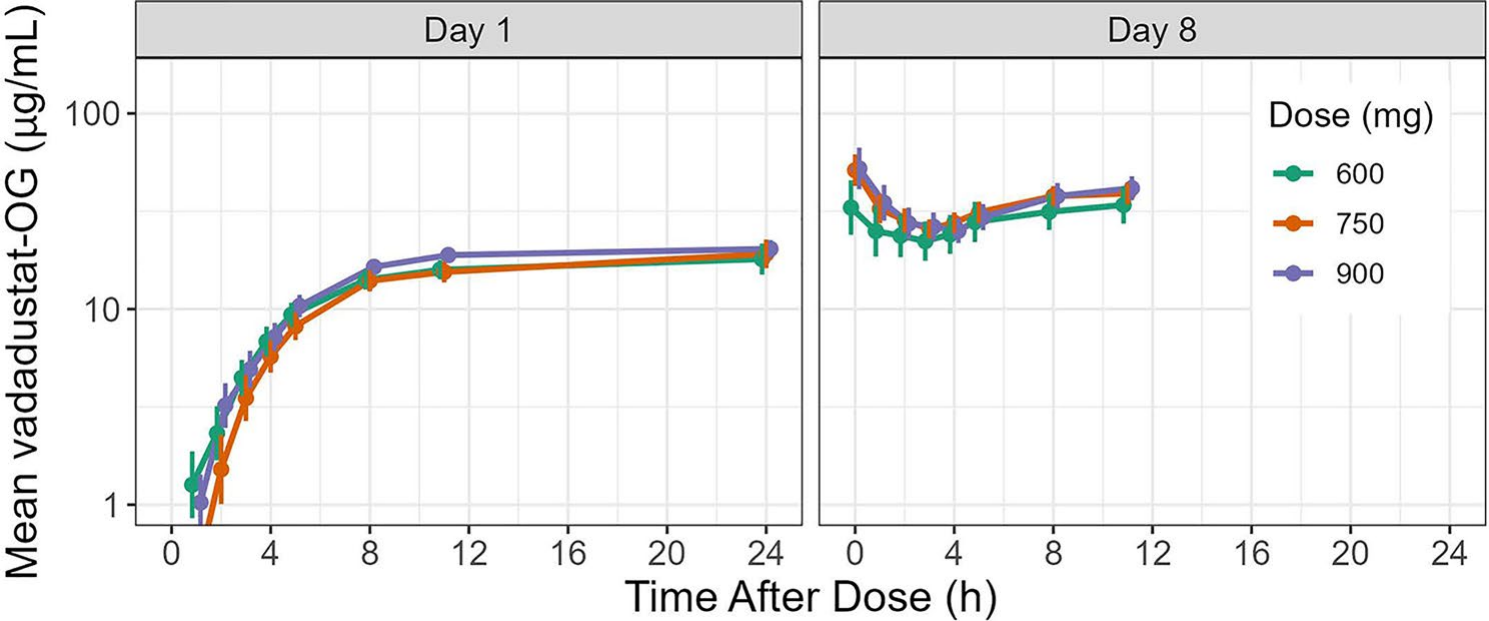
Mean plasma concentration-time profile for vadadustat-OG (µg/mL) on Day 1 and Day 8. Error bars indicate 95% CI; geometric mean values were used for vadadustat concentration calculations. h, hours

Mean concentrations of vadadustat-OG (the major vadadustat metabolite, which is an inactive and nontoxic) increased with time on Day 1 for all vadadustat groups, but no substantial difference across dose groups was observed (Fig. 3). However, an initial decline in vadadustat-OG concentration was observed at the early time points on Day 8 (Fig. 3). Based on a comparison of mean Day 1, 24-hour post-dose to pre-dose on Day 8, vadadustat-OG concentrations appeared to have increased 2.5- to 3.5-fold with multiple dosing in patients with DD-CKD (Fig. 3). Vadadustat-OG PK parameters are shown in Table 2.

**Table 2:** Summary of pharmacokinetic parameters for plasma vadadustat-OG concentration for vadadustat treatment groups

Parameter (Unit) Statistic	Vadadustat 600 mg, N = 12		Vadadustat 750 mg, N = 10		Vadadustat 900 mg, N = 13	
	Day 1	Day 8	Day 1	Day 8	Day 1	Day 8
C_max_ (μg/mL)						
n	12	12	10	10	13	13
Mean (SD)	23.2 (16.1)	53.6 (43.5)	25.5 (12.7)	68.1 (37.3)	23.5 (6.7)	72.3 (42.7)
AUC_0-24_ (h · μg/mL)						
n	12	12	10	10	13	13
Mean (SD)	357 (180.5)	1050 (960.0)	362 (132.2)	1143 (525.0)	382 (85.1)	1196 (604.9)
T_max_ (h)						
n	12	12	10	10	13	13
Median	23.1	0.0	23.7	0.0	11.0	0.0
Min, Max	10.0, 23.8	0.0, 10.8	5.0, 23.9	0.0, 10.6	10.0, 23.9	0.0, 11.0

Day 1 included the entire 24-hour sampling period. Day 8 included samples collected from pre-dose to 11 hours post-dose of Day 8 to calculate C_max_ and T_max_. In addition, Day 8 pre-dose was considered as result for the 24-hour time point to calculate AUC_0-24_ and t_1/2_. AUC_0-24_ corresponded to AUC_tau_, AUC_last_, and AUC_inf_ on Day 8. AUC_0-24_, area under concentration-time curve from time 0 to 24 hours; AUC_inf_, AUC from dosing to infinity; AUC_last_, AUC from dosing to last quantifiable concentration; AUC_tau_, AUC to end of the dosing period; C_max_, maximum observed plasma concentration; T_max_, time to maximum concentration.

### Pharmacodynamics results

**Fig. 4:**
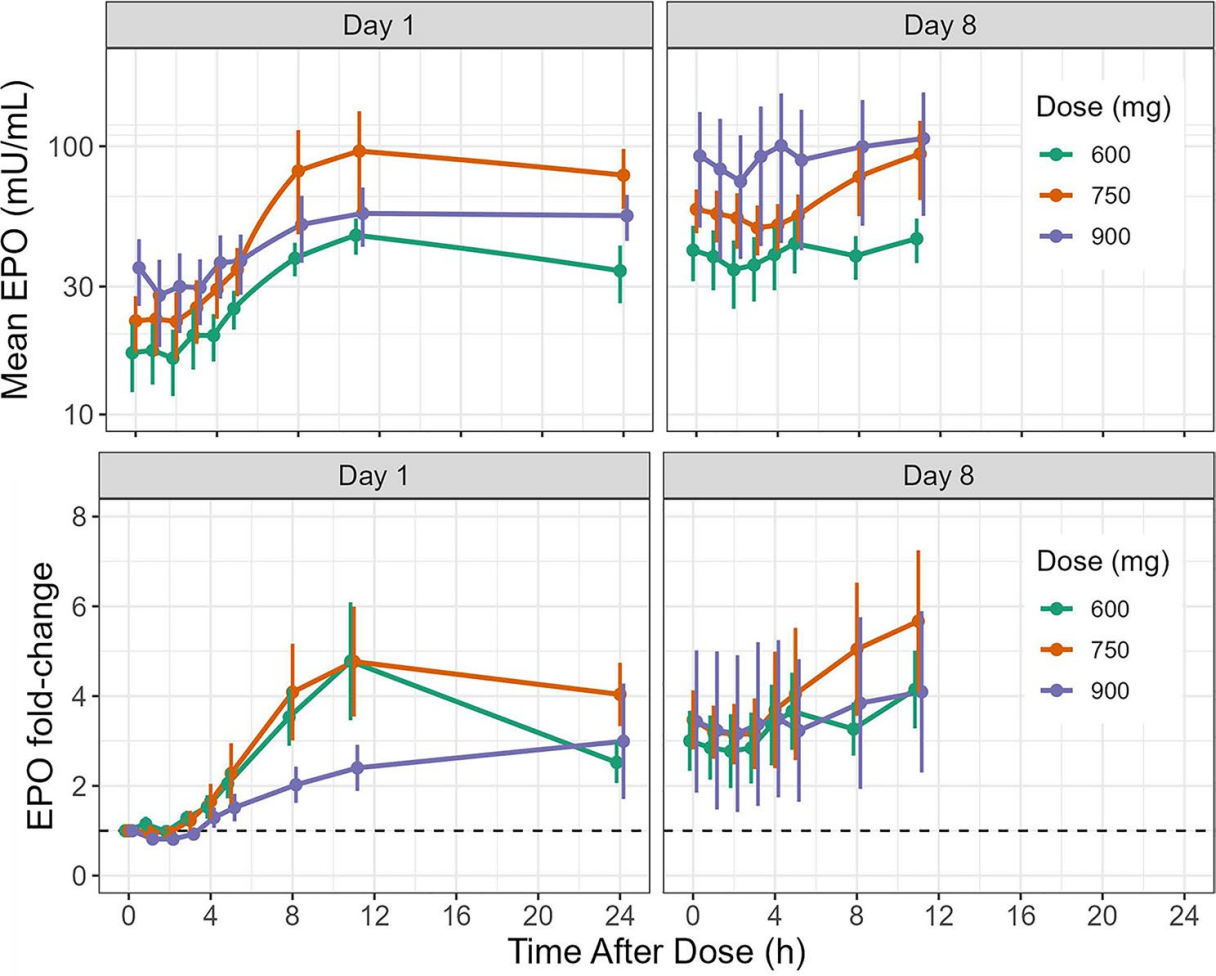
Mean and fold-change EPO concentration-time profile (mU/mL) in the vadadustat treatment group. Error bars indicate 95% CI. EPO, erythropoietin; h, hours

**Fig. 5:**
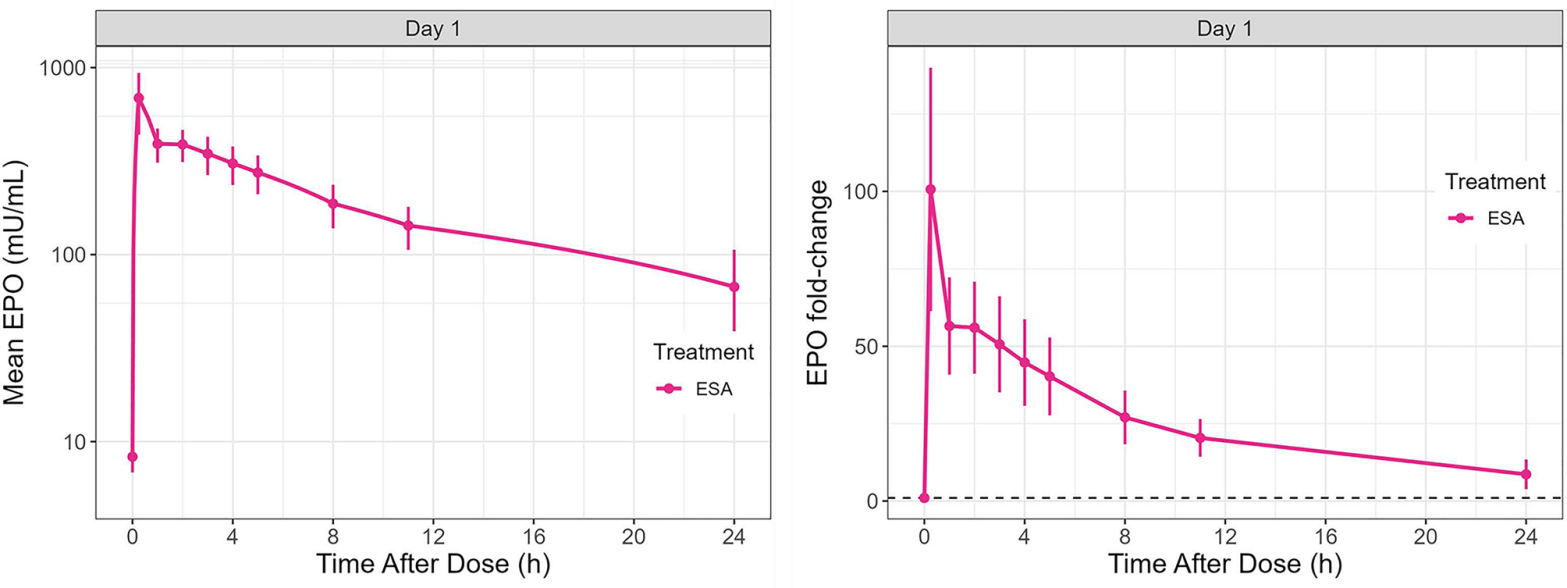
Mean and fold-change EPO concentration (mU/mL) in the ESA treatment group. Error bars indicate 95% CI. EPO, erythropoietin; ESA, erythropoiesis-stimulating agent; h, hours

Vadadustat increased plasma EPO concentrations in all participants, but a variable EPO response was observed in each dosing group (Fig. 4, Fig. A2). Following Day 1 dosing, the highest EPO increase was observed in the 750-mg vadadustat treatment group. There was a > 4-fold increase in peak EPO relative to the baseline in the 600- and 750-mg dosing groups on the first day. This contrasts with the 900-mg dosing group, where the EPO increase was just over 2-fold. EPO levels reached their peak 11 hours post-dose in all dosage groups. Following additional doses of vadadustat on Day 8, there was a > 5-fold increase in the 750-mg group, contrasting with the 4-fold increase observed in the 600- and 900-mg groups (Fig. 4). As depicted in Fig. 5, there was a 100-fold increase in EPO levels in the ESA group.

**Fig. 6.**
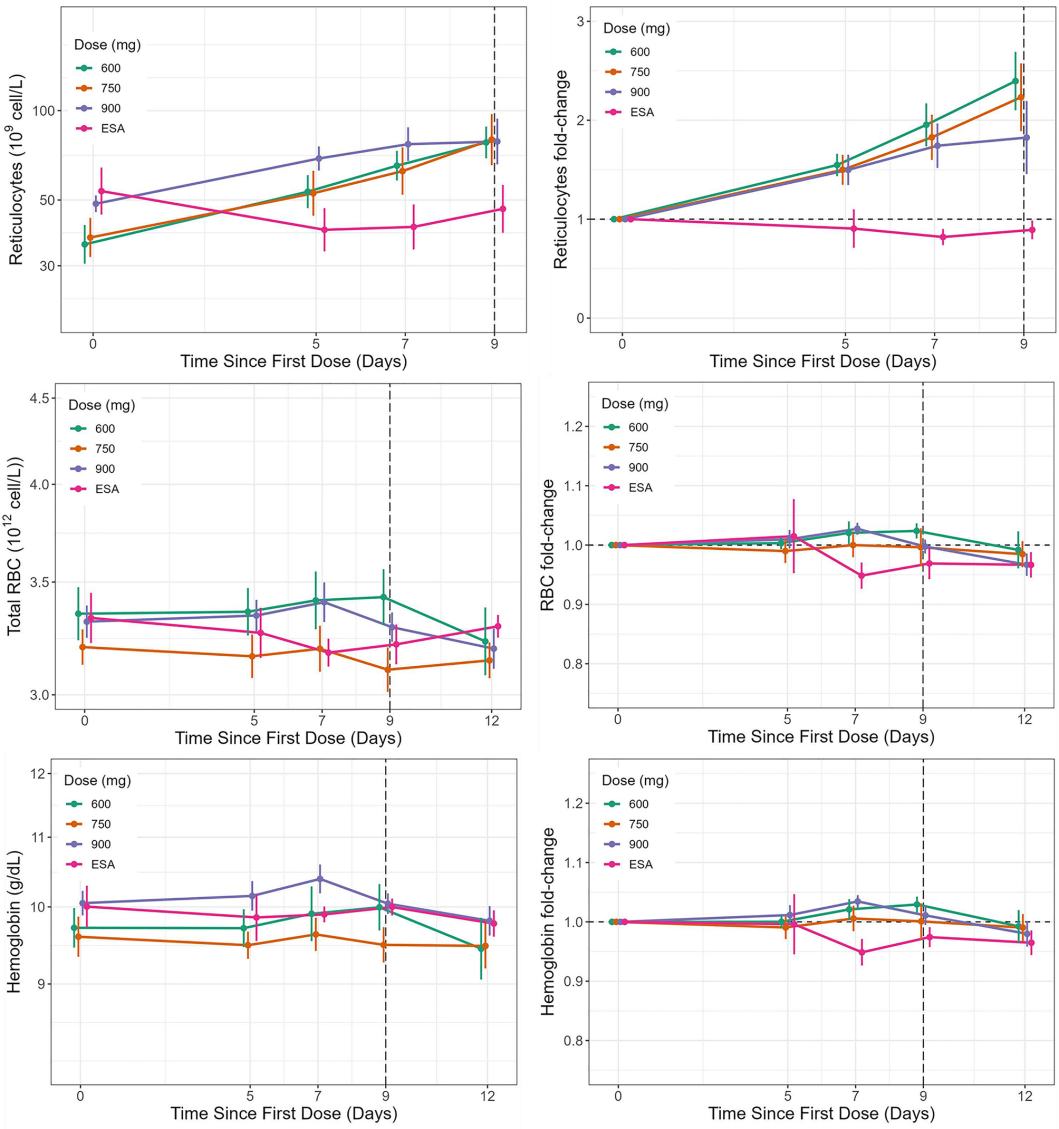
Mean and fold-change reticulocytes, hemoglobin, and RBC concentrations in the vadadustat and ESA treatment groups. Error bars indicate 95% CI. ESA, erythropoiesis-stimulating agent; h, hours; RBC, red blood cell

In relation to other PD parameters (Fig. 6), all vadadustat dose groups showed an approximately 2-fold increase in mean reticulocyte count on Day 5, which continued to rise through the final on-treatment assessment 9 days after the initial dose, yet there was no significant difference observed between the various doses. In contrast, the reticulocyte count exhibited minimal change in the group receiving the ESA treatment (Fig. 6). Mean hemoglobin and RBC levels remained unchanged relative to baseline, and no significant changes were observed in both the vadadustat and ESA treatment groups.

### Safety and tolerability

In the vadadustat treatment groups, 20 TEAEs were reported by 11 patients, while no TEAEs were reported in the ESA treatment group (Table 3). At least 1 TEAE was experienced by 3 (25.0%), 3 (25.0%), and 5 (38.5%) patients in the vadadustat 600-, 750-, and 900-mg treatment groups, respectively. One patient in each of the groups experienced a drug-related TEAE, and a treatment-emergent serious AE was reported by 1 patient in both the vadadustat 600- and 750-mg treatment groups. In the 600-mg vadadustat treatment group, a TEAE of nausea was considered to be drug related but nonserious. In the 750-mg vadadustat treatment group, 1 patient experienced decreased appetite and later showed elevations in ALT/AST enzymes, which was considered to be drug related. However, no abnormal values of bilirubin or alkaline phosphatase were observed in this patient during this period. In the 900-mg group, a TEAE of diarrhea was considered to be drug related but nonserious. All TEAEs reported in the vadadustat treatment groups were either mild or moderate in intensity, and there were no TEAEs leading to treatment discontinuation or death. Diarrhea was experienced by 3 patients in the vadadustat treatment groups (2 in the 750-mg group and 1 in the 900-mg group). No other TEAE was experienced by > 1 patient in the vadadustat treatment group.

**Table 3:** Overall summary of treatment-emergent adverse events

Category	Vadadustat 600 mg N = 12 n (%)	Vadadustat 750 mg N = 12 n (%)	Vadadustat 900 mg N = 13 n (%)	Vadadustat Total N = 37 n (%)	IV ESA N = 7 n (%)
Number of TEAEs	8	6	6	20	0
Patients with any TEAEs	3 (25.0)	3 (25.0)	5 (38.5)	11 (29.7)	0
Patients with any drug-related TEAEs	1 (8.3)	1 (8.3)	1 (7.7)	3 (8.1)	0
Patients with any severe TEAEs	0	0	0	0	0
Patients with any treatment-emergent SAEs	1 (8.3)	1 (8.3)	0	2 (5.4)	0
Patients with any TEAEs leading to study treatment discontinuation	0	0	0	0	0
Patients with any TEAEs leading to death	0	0	0	0	0

IV ESA: darbepoetin alfa or epoetin alfa. A TEAE was an AE that began (or a pre-existing AE that worsened) after receiving study drug and within 30 days of the last dose. ESA, erythropoiesis-stimulating agent; IV, intravenous; SAEs, serious adverse events; TEAEs, treatment-emergent adverse events

## Discussion

Vadadustat is an orally administered HIF-PHI that has been developed to treat anemia associated with CKD [15, 21]. Pivotal phase 3 trials included a vadadustat starting dose of 300 mg with a maximum dose of 600 mg [11, 12]. Here, we report a phase 1b study with a goal to evaluate the pharmacokinetics, pharmacodynamics, safety, and tolerability of vadadustat 600, 750, or 900 mg daily in DD-CKD. An active control group continued prior treatment with IV darbepoetin alfa or epoetin.

Vadadustat exhibited a dose-dependent increase in AUC, but consistently similar CL/F values across doses, a result that aligns with prior findings [19]. The C_max_ and AUC ratios for vadadustat on Day 8 vs Day 1 were < 2, indicating modest accumulation of the parent drug. An initial decrease in vadadustat-OG concentration was observed on Day 8 in the first 4 hours after dosing. These findings are consistent with the delay of formation of the metabolite and its accumulation in plasma in DD-CKD patients. Previous studies have indicated that vadadustat was detected in the urine as vadadustat-OG in healthy volunteers; however, it appears that there are multiple simultaneous elimination pathways in patients with CKD aligning with nonclinical absorption, distribution, metabolism, and excretion data that propose a combination of renal and hepatic routes for the elimination of vadadustat. Importantly, although both PK collection days coincided with hemodialysis sessions, previous clinical studies have demonstrated that hemodialysis does not have a significant effect on the exposures of vadadustat or its metabolite (data not shown). Therefore, the observed PK profiles are unlikely to be confounded by hemodialysis-related elimination of vadadustat or its metabolite. In addition, the PK observed in this study are broadly similar with those reported in earlier clinical studies, with expected variability attributable to differences in formulation and bioanalytical methods used across the clinical program. Furthermore, previous studies have demonstrated that patients with CKD experience a modest increase in mean half-life and a decrease in CL/F. As eGFR decreases, CL/F is reduced and terminal half-life is prolonged, indicating a renal component to vadadustat elimination. These findings suggest that patients with CKD experience a longer half-life and greater exposure to vadadustat at a given dose compared with healthy volunteers [19].

Vadadustat triggered a variable EPO response. After the first day of dosing, the largest increase in EPO was observed in the group treated with 750 mg of vadadustat, primarily due to a single-patient surge in EPO levels. The data suggest that there was no clear dose-response relationship in EPO levels following vadadustat treatment. As expected, there was a much higher increase in EPO level activity in the ESA cohort due to exogenous IV ESA administration.

All vadadustat dose groups showed a continuous increase in the mean reticulocyte count until the end of treatment; this result is consistent with previous findings [17]. Despite this trend, there were no significant differences observed between doses due to the variability of the data. Conversely, in the ESA-treated group, there were no significant changes in the reticulocyte count. This can be attributed to the absence of a washout period in this group, which suggests there was no restimulation of reticulocyte production, unlike in the vadadustat treatment groups. Mean hemoglobin and RBC concentrations did not show clinically meaningful changes in any of the vadadustat or ESA groups during the 10-day treatment due to the short treatment duration.

The primary TEAEs in the vadadustat groups, primarily GI related, could be attributed to the use of an oral dosage form (vadadustat) compared with an injectable ESA. There were no deaths or discontinuations from study treatment due to AEs.

### Conclusions

This study characterizes the exposure, biomarker response, and safety profile of vadadustat at doses of 600, 750, and 900 mg in patients with CKD receiving dialysis. This marks the first instance of the higher doses, specifically 750 and 900 mg, being explored within a patient population. These findings will contribute to the development of higher-dose regimens for further investigation in clinical studies.

## Electronic supplementary material

Below is the link to the electronic supplementary material.

Supplementary Material 1

## Data Availability

Proposal requests for access to full trial protocol and original data should be sent to medicalinfo@akebia.com. Deidentified patient-level data will be available 12 months after US and EU approval to qualified researchers with an appropriate research proposal. The research proposal is subject to review by an IRB with final approval by Akebia Therapeutics, Inc.

## Abbreviations

AEs: Adverse events
ALT: Alanine aminotransferase
AUC: Area under the plasma concentration time curve
AUC_0-last_: AUC from dosing to last quantifiable concentration
AUC_0-inf_: AUC from dosing to infinity
AUC_0-24_: Time 0 to 24 hours
AUC_tau_: AUC to the end of the dosing period
AST: Aspartate aminotransferase
CKD: Chronic kidney disease
CL/F: Oral clearance
C_max_: Maximum plasma concentration
DD: Dialysis-dependent
EPO: Erythropoietin
ESA: Erythropoiesis-stimulating agent
GI: Gastrointestinal
HIF-PHI: Hypoxia-inducible factor prolyl hydroxylase inhibitor
IV: Intravenous
Kt/Vurea: ‘K’ is dialyzer urea clearance, ‘t’ is total dialysis session time, and ‘V’ is volume of distribution of urea
NDD: Non-dialysis-dependent
PD: Pharmacodynamic
PK: Pharmacokinetic
RBC: Red blood cell
t_1/2_: Half-life
TEAE: Treatment-emergent adverse event
T_max_: Time to maximum concentration
Vz: Volume of distribution
