# Supplementary material for: A randomized trial to evaluate the pharmacokinetics, pharmacodynamics, and safety of vadadustat in patients with anemia associated with chronic kidney disease receiving hemodialysis
Source: BMC Nephrol. 2025 Aug 11;26:453. doi: 10.1186/s12882-025-04367-x (PMC12341096; doi:10.1186/s12882-025-04367-x)
Supplement: Supplementary file 1 — Supplementary Material 1 [file 12882_2025_4367_MOESM1_ESM.docx]

**Additional file 1 for:**

**A Randomized Trial to Evaluate of the Pharmacokinetics, Pharmacodynamics, and Safety of Vadadustat in Patients With Anemia Associated With Chronic Kidney Disease Receiving Hemodialysis**

Navarro-Gonzales P, et al.

**Figure A1. Patient Disposition**

**
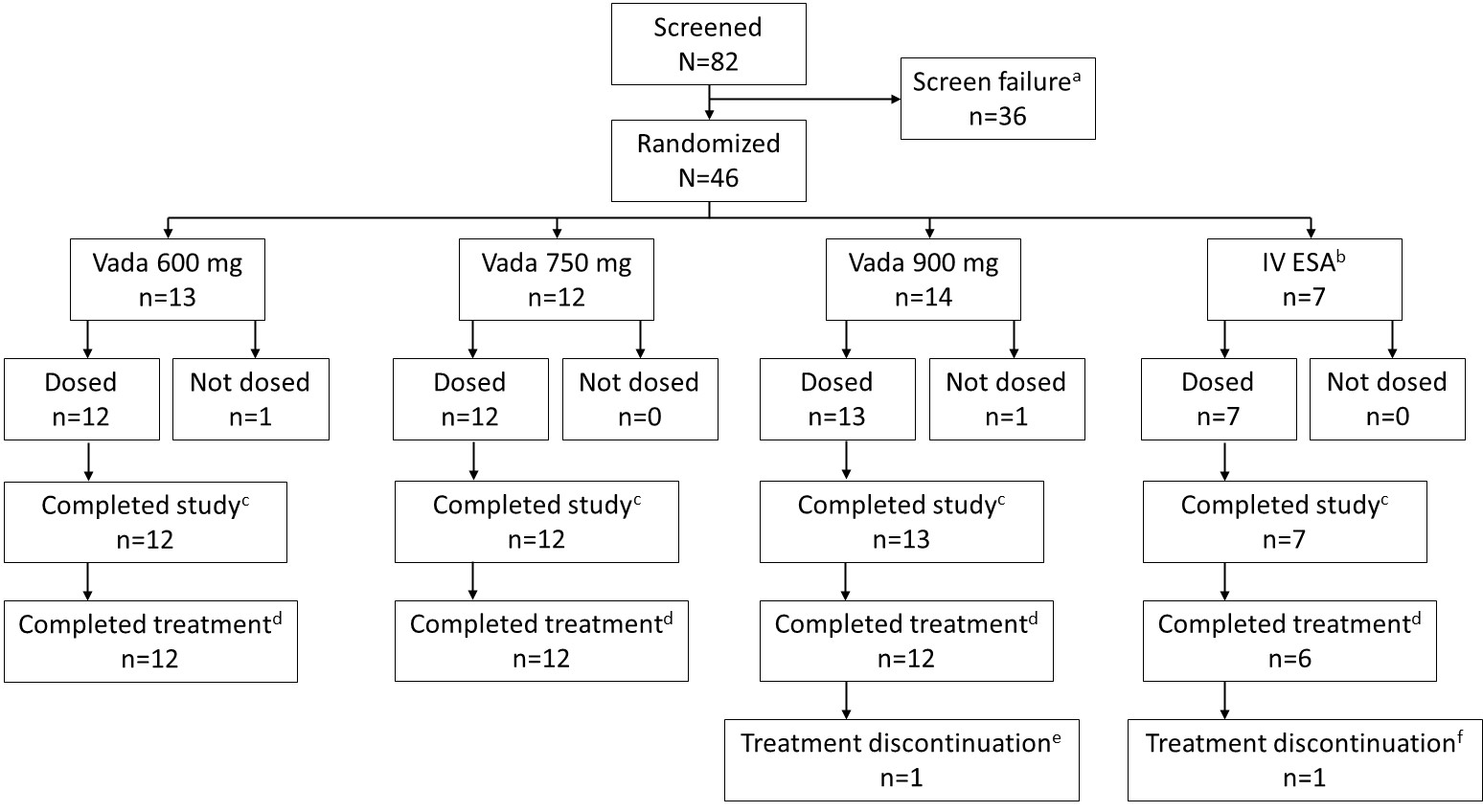
**

^a^ Patients who did not meet eligibility criteria. ^b^ Darbepoetin alfa (n=1) or epoetin alfa (n=6). ^c^ Patients who completed the 30-day follow-up call post last dose, irrespective of the number of doses of study drug received. ^d^ Patients who completed the treatment day 10. ^e^ One patient was not dosed on day 10 due to rise in Hb level >12.0 g/L (identified on day 8); this patient did not complete treatment, but did complete the study, and was included in the safety and PK populations. ^f^ One patient dosed with darbepoetin alfa met early discontinuation criteria due to a rapid rise in Hb (>1.5 g/L over baseline, identified on day 6); this patient did not complete treatment, but did complete the study, and was included in the safety and PK populations.

ESA: erythropoiesis-stimulating agent; Hb, hemoglobin; IV: intravenous; vada: vadadustat.

**Figure A2. Geometric mean and fold-change EPO concentration-time profile (mU/mL) in the vadadustat treatment group**


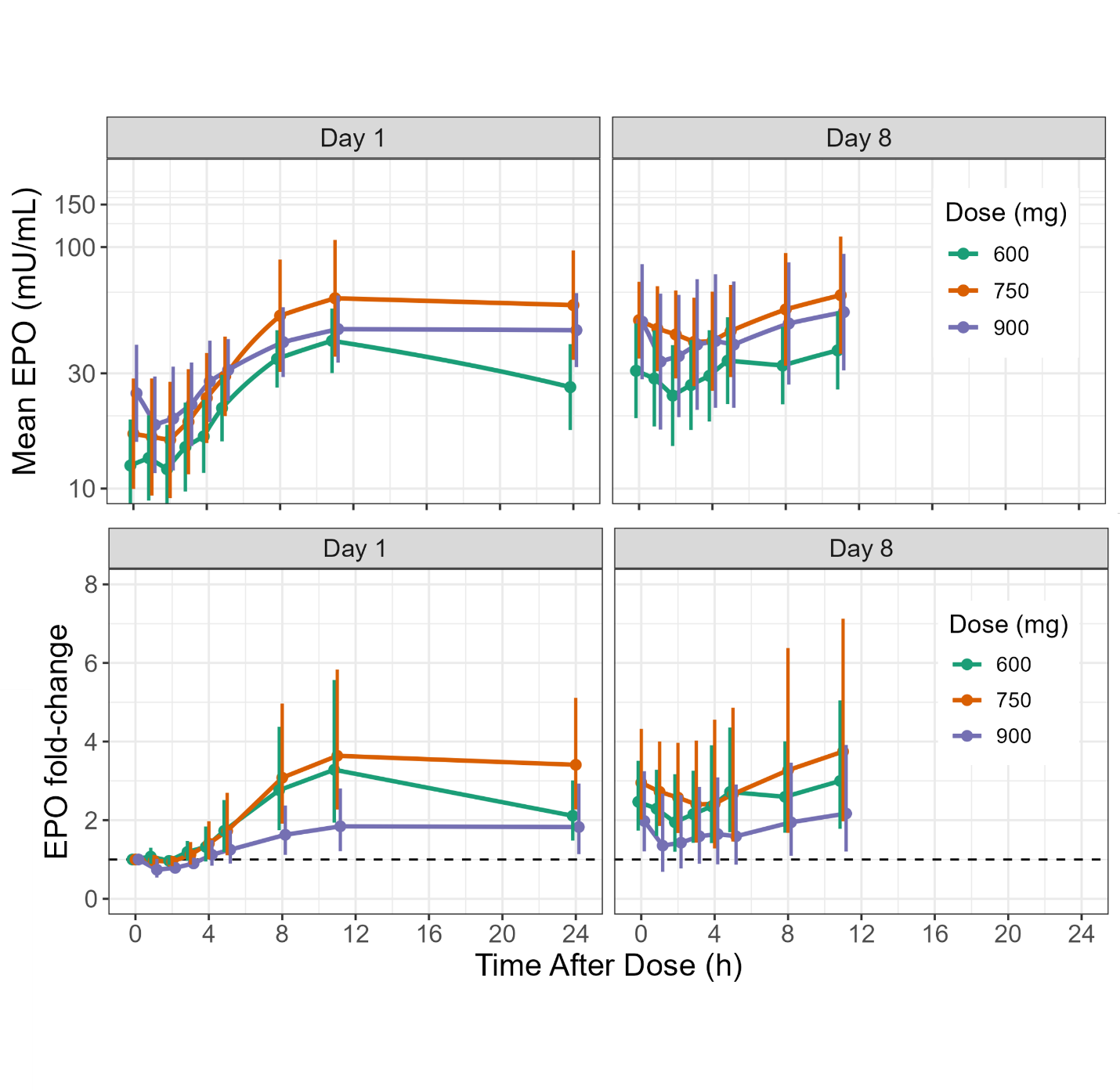


Error bars indicate 95% CI.

EPO, erythropoietin; h, hours.

**Table A1. Summary of demographic characteristics (randomized population)**

| Characteristics | Vadadustat 600 mg N=13 | Vadadustat  750 mg N=12 | Vadadustat  900 mg N=14 | Vadadustat  Total N=39 | IV  ESA N=7 |
| --- | --- | --- | --- | --- | --- |
| Age mean (SD) | 60.8 (10.54) | 54.0 (12.80) | 55.1 (13.38) | 56.7 (12.37) | 58.1 (7.65) |
| Female, n (%) | 9 (69.2) | 5 (41.7) | 5 (35.7) | 19 (48.7) | 3 (42.9) |
| Ethnicity, n (%)  Hispanic or Latino  Not Hispanic or Latino  Not reported /unknown | 0  13 (100)  0 | 0  12 (100)  0 | 2 (14.3)  12 (85.7)  0 | 2 (5.1)  37 (94.9)  0 | 1 (14.3)  6 (85.7)  0 |
| Race, n (%)  American Indian, Native Hawaiian, and  Other Pacific Islander  Asian  Black  White | 0  1 (7.7)  12 (92.3)  0 | 0  0  11 (91.7)  1 (8.3) | 0  0  10 (71.4)  4 (28.6) | 0  1 (2.6)  33 (84.6)  5 (12.8) | 0  0  5 (71.4)  2 (28.6) |
| Height (cm), n  Mean (SD)  Median (min, max) | 12  168.4 (10.9)  167.3 (155.0, 188.0) | 12  172.9 (9.1)  175.1 (154.9, 185.4) | 13  167.8 (10.5)  170.2 (152.0, 182.9) | 37  169.7 (10.2)  170.2 (152.0, 188.0) | 7  172.0 (13.1)  170.2  (152.4, 190.5) |
| Weight (kg), n  Mean (SD)  Median (min, max) | 12  74.6 (18.9)  72.4 (43.5, 109.8) | 12  100.4 (32.2)  90.9 (63.1, 166.5) | 13  88.3 (31.8)  88.2 (58.6, 167.7) | 37  87.8 (29.6)  85.2 (43.5, 167.7) | 7  93.9 (23.2)  92.5 (69.4, 142.1) |
| Baseline ESA dose, U/kg/wk  Darbepoetin alfa,^a^ n  mean (SD)  Epoetin alfa,^b^ n  mean (SD) | 8  58.13 (32.5)  4  2150 (1852) | 5  58.00 (29.7)  7  2500 (1859) | 6  55.83 (38.0)  7  3814 (3312) | 19  57.37 (31.8)  18  2933(2498) | 1  100.0 (NA)  6  1766 (930.9) |

^a^One time per week. ^b^Three times per week.

IV ESA: darbepoetin alfa or epoetin alfa; 6 patients were dosed with epoetin alfa, and 1 patient was dosed with darbepoetin alfa.
ESA, erythropoiesis-stimulating agent; IV, intravenous.

**Table A2. Analysis Populations**

| **Category** | **Vadadustat 600 mg, n (%)** | Vadadustat 750 mg, n (%) | Vadadustat  **900 mg, n (%)** | Vadadustat  **Total, n (%)** | IV ESA, n (%) |
| --- | --- | --- | --- | --- | --- |
| Randomized Population^a^ | 13 | 12 | 14 | 39 | 7 |
| Safety Population^b^ | 12 (92.3) | 12 (100) | 13 (92.9) | 37 (94.9) | 7 (100) |
| PK Population^c^ | 12 (92.3) | 10 (83.3) | 13 (92.9) | 35 (89.7) | 7 (100) |

^a^ The randomized population was defined as all randomized patients; analyses of this population was based on the randomized treatment group. ^b^ The safety population was defined as all patients who received at least one dose of study drug; this population was analyzed based upon the actual treatment received. ^c^ The PK population included all patients who completed the study without any protocol deviation(s) that would impact the study results, and for whom the PK profile could be adequately characterized. In the 750 mg vadadustat dose group, one patient had inadvertent ESA administration and another patient had the entire concentration profile below quantitation limit for every vadadustat PK sample and therefore, these two patients were excluded from the PK population.

Percentages are based on all randomized patients; IV ESA: darbepoetin alfa or epoetin alfa.

ESA: erythropoiesis-stimulating agent; IV: intravenous; PK: Pharmacokinetic.
